# Heart Failure in Older Patients: An Update

**DOI:** 10.3390/jcm14061982

**Published:** 2025-03-14

**Authors:** Massimo Montalto, Federica D’Ignazio, Sara Camilli, Silvino Di Francesco, Marco Fedele, Francesco Landi, Antonella Gallo

**Affiliations:** 1 Department of Geriatrics, Orthopedics and Rheumatology, Fondazione Policlinico Universitario “A. Gemelli”, IRCCS, 00168 Rome, Italy; massimo.montalto@unicatt.it (M.M.); francesco.landi@unicatt.it (F.L.); 2 Department of Geriatrics, Orthopedics and Rheumatology, Università Cattolica del Sacro Cuore, 00168 Roma, Italy; federica.dignazio@guest.policlinicogemelli.it (F.D.); sara.camilli01@icatt.it (S.C.); silvino.difrancesco01@icatt.it (S.D.F.); marco.fedele01@icatt.it (M.F.)

**Keywords:** heart failure, elderly, older patients, frailty, cardiovascular aging, palliative care

## Abstract

Heart failure (HF) is a clinical syndrome with high incidence and prevalence and high morbidity and death rate, even in the short term, representing a serious public health issue, mainly in older people. It is a growing cause for hospital admission in this age group, being frequently associated with several comorbidities, further aggravating the disease’s course. Moreover, older HF patients are usually affected by clinical conditions, like frailty, malnutrition, and cachexia, which significantly impact the overall management of HF and need to be properly identified and treated. Diagnosing and managing HF in older patients may be very complicated and challenging. Although specific data on treatment of both acute and chronic HF in older subjects are limited and mainly extrapolated from large-scale clinical trials, the standard pharmacological management may be considered well-tolerated and generally safe. In any case, a personalized and tailored approach is mandatory and is based on severity of comorbidities, overall status, and prognosis, above all in frailer and more comorbid subjects, due to the higher rate of drug interactions, side effects, and therapy discontinuation in this population. In this scenario, palliative care has become a fundamental part of HF management in the elderly in order to improve their care and the quality of life. Moreover, an increasing number of promising pharmacological options deserve further investigation in order to support clinicians in optimizing management of comorbid and frailer patients. In this work, we provide detailed and updated insight into clinical, therapeutic, and prognostic features of both acute and chronic HF in the older population.

## 1. Introduction

Heart failure (HF) is a clinical syndrome with symptoms and/or signs caused by a structural and/or functional cardiac abnormality and corroborated by elevated natriuretic peptide levels and/or objective evidence of pulmonary or systemic congestion [1]. With high incidence and prevalence and high morbidity and death rate even in the short term, HF represents a serious public health issue [1,2,3]. The prevalence of HF gradually rises with age, reaching around 20% in adults over 75 [4,5]. As 17% of the world’s population is expected to be over 85 by 2050 [3], HF has clearly become a significant burden for the global health system.

Different mechanisms have been supposed to contribute to the development of age-related cardiovascular diseases, including HF, such as oxidative stress from mitochondrial dysfunction, chronic inflammation (particularly in obese patients), immune dysfunction, and low cardiomyocyte renewal rate due to a limited capacity for cardiac regeneration [6,7,8,9].

Biological, hormonal, and socio-environmental factors also contribute to HF pathogenesis, mainly in post-menopausal women. Besides having smaller cardiac volumes, women exhibit higher systolic and diastolic left ventricular (LV) stiffness compared to men, which intensifies with aging [10], and, on the other side, lower chronotropic and contractile reserve, contributing to exercise intolerance [10]. Moreover, the post-menopausal reduction in estrogen levels (which plays a protective role in the cardiovascular system) and concomitant increase in testosterone contribute to the activation of the renin-angiotensin-aldosterone system, with a consequent rise in reactive oxygen species and decrease in nitric oxide, leading to endothelial dysfunction and microvascular impairment typical of HF with preserved contractility [11]. This dysfunction exacerbates microvascular abnormalities, such as capillary rarefaction, which diminishes the ability of blood vessels to respond effectively to changes in blood flow, contributing to impaired diastolic function and increased left ventricular stiffness.

In addition to hormonal factors, several comorbidities prevalent in elderly women, such as hypertension, obesity, and diabetes, further increase the risk of developing heart failure with preserved ejection fraction (HFpEF). Hypertension, which induces left ventricular hypertrophy, is more prevalent among female HF patients compared to their male counterparts, representing 50% and 40%, respectively [10]; diabetes mellitus increases the risk of HF fivefold in women compared to 2.4 times in men, affecting approximately 45% of patients with HFpEF [10]; obesity affects more women than men globally and impacts up to half of patients with HFpEF [10,11,12]; autoimmune diseases, which are more common in women, are associated with diastolic dysfunction, as seen in rheumatoid arthritis [10,12]. Moreover, iron deficiency, more common in women, is another critical factor that can worsen cardiac function by reducing myocardial efficiency and increasing ventricular stiffness, contributing to diastolic dysfunction. In conclusion, the combination of endothelial dysfunction, impaired angiogenesis, and increased left ventricular stiffness are crucial contributors to the pathogenesis of HFpEF, which represents the typical subtype of HF in women [10,11,12].

As a consequence, older patients visiting an emergency department (ED) with acute decompensating heart failure (ADHF) are frequently female and have an established history of HFpEF [13,14,15,16], most often of hypertensive etiology [17,18]. In any case, older HF patients usually present with multiple coexisting conditions that require concomitant careful management, including atrial fibrillation, dementia, hypertension, valvular heart disease, and kidney dysfunction [14,19,20]. Conversely, conditions such as diabetes and coronary artery disease appear to be less prevalent compared to younger subjects, as these cardiovascular comorbidities, with the exception of hypertension, typically follow a non-linear progression [16,21].

ADHF is one of the leading causes of hospitalization in patients over 65 years old in Western nations [22,23], and advanced age is one of the most important negative prognostic factors for mortality [13,24]. Most of all, HF may significantly further aggravate the concomitant disease’s course, and it is commonly associated with other age-related conditions, like frailty, malnutrition, and cachexia [5], seriously impacting the overall prognosis.

Frailty, in particular, is characterized by a decline in physiological reserve under stress conditions, making the patient more vulnerable to adverse events [25] and to endogenous and/or exogenous stressors [26]. Frailty is linked to a 1.5 overall hazard risk for hospitalization and death in chronic heart failure [27], as well as a higher 30-day mortality rate in acute situations [28]. Additionally, in individuals with HF, frailty may be observed in up to 45% of cases, with a significantly increased risk of functional decline, hospitalization, longer hospital stays, and mortality [22,26]. Moreover, literature shows that frailty is linked to increased left ventricular stiffness, subclinical LV remodeling, and diastolic dysfunction with a bidirectional relationship [29]. Similarly, malnutrition, involving up to two-thirds of HF patients, is closely linked to one-year mortality due to appetite loss, malabsorption, and a catabolic condition [30]. In addition, cachexia, defined as an aberrant catabolic/anabolic balance, is commonly observed in several chronic conditions [31]. It affects approximately 10% of CHF patients and may impact the general prognosis [32].

Sarcopenia is considered one of the main causes of decreased physical performance and impaired cardiorespiratory fitness in older individuals with HF and contributes significantly to poor outcomes. However, muscle wasting in HF presents different pathogenic mechanisms in sarcopenic and cachectic states [33,34]. Heart failure can lead to sarcopenia through shared mechanisms such as hormonal changes, malnutrition, and physical inactivity, like alterations in the ubiquitin proteasome system, myostatin signaling, and apoptosis. Interestingly, in sarcopenia, the heart muscle tends toward pathological hypertrophy [33,34].

Studies have found that 43% of HF patients experience cognitive impairment, involving attention, language, memory, and executive functions. Although cognitive dysfunction is commonly associated with HF, the prevalence varies between HF phenotypes, with some studies showing a higher frequency in HFrEF compared to HFpEF [35]. The mechanisms behind cognitive decline in HF start from shared risk factors like hypertension, diabetes, transient ischemic attack, or stroke. These mechanisms are related to reduced blood flow and may also involve inflammation and increased sympathetic nervous system activity [36,37,38]. In HFpEF, cognitive decline is more likely related to comorbid vascular risk factors such as diabetes, hypertension, and atherosclerosis. Obesity and sleep-disordered breathing, commonly found in HF patients, also contribute to cognitive decline, increasing the risk of stroke due to systemic inflammation and of abnormal cardiac rhythms, like atrial fibrillation [36,37]. Genetic variations associated with Alzheimer’s disease (AD) have been found in HF patients, and biomarkers of neurodegeneration have been linked to cardiac dysfunction. For example, intramyocardial deposits of amyloid-β40 and β42, commonly seen in neurodegeneration, as well as in diastolic dysfunction, have been observed in AD patients with HF [38]. 

Based on these elements, beyond its poor prognosis and significant residual disability after hospital discharge, some authors consider HF a true geriatric syndrome [5]. Therefore, a multidisciplinary and holistic approach has univocally been invocated for older patients with HF, starting from the onset of the disease to its terminal stage [22,39]. Both CHF and ADHF may exhibit typical features in the elderly, above all in terms of clinical presentation, laboratory findings, and relevant prognostic factors [22,24].

In this work, we summarize the peculiarity and concerns about HF management in advanced age, according to both current guidelines and available clinical practice and real-world data.

## 2. Diagnostic Challenges

According to the current European guidelines [22], the diagnosis of HF is based on suggestive symptoms, an increase in natriuretic peptides, and echocardiographic findings.

The primary symptom of heart failure (HF) is dyspnea, a clinical manifestation indicative of elevated pulmonary pressure, reflecting significant cardiovascular dysfunction. Nevertheless, signs and symptoms often exhibit limited clinical diagnostic sensitivity and specificity in the elderly [5,40,41]. Atypical presentations, such as confusion, somnolence, anorexia, and decreased physical activity, may be much more frequent than expected in these subjects (Table 1) [5,40,41].

In this regard, a 2013 post-hoc study showed a significant difference in signs and symptoms at presentation between younger (<65 years) and older patients [41]; younger patients showed less dyspnea, as indicated by a lower New York Heart Association (NYHA) functional class, and less frequently had peripheral edema and/or rales. In contrast, they often presented with signs of paroxysmal nocturnal dyspnea, elevated jugular venous pressure, and hepatomegaly [41].

Besides clinical presentation, older patients are usually less likely to realize and report to physicians the occurrence of symptoms compatible with HF, leading to delayed responses during HF exacerbations [42]. In addition, early and subtle symptoms of HF, such as fatigue and weight gain, are often not quickly detected and are frequently misattributed to aging or comorbid conditions [42].

In addition, laboratory exams may reveal peculiar findings in elderly HF patients, as routine tests often show reduced hemoglobin (Hb) levels and impaired serum sodium levels, both in term of hyponatremia, due to hemodilution by fluid retention-congestion or loop-diuretics therapy, and of hypernatremia due to excessive sodium retention mechanisms. Moreover, blood urea nitrogen (BUN) and creatinine levels typically rise, and a drop in the estimated glomerular filtration rate (eGFR), better reflecting renal reserve decrease with age, [14,16,20], is commonly observed, mainly in cases of ADHF.

The determination of N-terminal pro-B-type natriuretic peptide (NT-proBNP) levels is a well-recognized valuable tool in the diagnostic process due to its high negative predictive value, both in the chronic and the acute setting. The ESC 2021 guidelines establish a NT-proBNP cut-off of 125 pg/mL in non-acute conditions and 300 pg/mL in acute settings for a probable HF diagnosis, without distinction for age or comorbidities [22]. However, in clinical practice, NT-proBNP dosage can be influenced by numerous comorbidities, such as atrial fibrillation, renal insufficiency, COPD, infections, and anemia [22], making its interpretation more complex [35,36,37,38].

In the latest two decades, different studies have identified different cut-off values of NT-proBNP levels according to age, ranging from a cut-off of 450 pg/mL for patients aged <50 years to about 2000 pg/mL in patients aged 75–80 years old [43,44,45,46] (Table 2). Finally, in a more recent study involving 12,071 patients over 75 years old (mean age 84 years), hospitalized for acute dyspnea, for NT-proBNP dosage within 48 h of ED admission, the optimal NT-proBNP threshold values for a final diagnosis of ADHF changed according not only to age but also to comorbidities [46] (Table 2). Therefore, a global assessment is mandatory in older patients visiting an ED for suspicion of HF, as a lonely laboratory parameter may be influenced by numerous variables.

Furthermore, since in older patients the absolute BNP value can be influenced by several factors, it may be more useful throughout follow-up to evaluate the trend of natriuretic peptide levels over time rather than the absolute value itself. It has been suggested that in elderly patients, a BNP variation greater than 29% over a 12-month follow-up period shows good specificity and sensitivity in predicting an episode of decompensated heart failure [47]. Although this biomarker has a recognized role in guiding therapy for chronic heart failure in younger patients, this does not appear to be the case for elderly patients, showing less effectiveness in terms of reducing hospitalizations and mortality [48,49,50]. Due to these limitations in the use of natriuretic peptides, over the past few years, scientific attention has shifted towards new potential biomarkers useful for the management of heart failure in elderly patients.

Interest has been recently growing related to the soluble suppressor of tumorigenicity 2 (sST2), a marker of cardiomyocyte stress and fibrosis, which, if used alongside conventional biomarkers, can provide a more reliable indication of inflammation status, myocardial stress, and the degree of fibrosis in the geriatric population affected by both HFpEF and HFrEF, as it is less influenced by age and renal insufficiency [51,52,53]. Ravi H. Parikh et al. conducted an observational study involving 3915 community-dwelling older patients (mean age 72.7 ± 5.5 years) and demonstrated that higher levels of sST2 were associated with an increased risk of incident HF and cardiovascular death [54]. However, although the prognostic value is significant in patients younger than 65 years, multivariable analysis models showed that in patients older than 65 years, the association between sST2 levels and the increased risk of heart failure and cardiovascular mortality is more modest, likely due to the presence of concomitant non-cardiovascular inflammatory diseases affecting the sST2 levels. In addition, Taheri reported that in 238 elderly patients (median age of 85 years) admitted to the ED for acute dyspnea, NT-proBNP showed the best diagnostic accuracy for ADHF compared to CD146, hs-cTnI, galectin-3, and ST2 [55].

Echocardiography is recommended as a key investigation tool for assessing cardiac function, providing morphological, structural, and hemodynamic insights. In a historical context marked by a significant increase in diagnoses of HFpEF, it is extremely important to identify risk factors and morphological-structural abnormalities before symptom development and to prevent adverse outcomes. According to the European Association of Cardiovascular Imaging/American Society of Echocardiography (EACVI/ASE) [10,56,57,58,59,60,61], the main echocardiographic pathological findings in the elderly involve the concentric remodeling of the left ventricle and the diastolic dysfunction [10], mainly characterized by an increase in left atrial volume and elevated filling pressures over time due to chronic disease [58], left ventricular hypertrophy [59,60], and age-dependent reduction of left ventricular longitudinal systolic strain [58]. Moreover, mitral annulus calcification (MAC), usually associated with a higher risk of ischemic stroke [60], aortic valve sclerosis (with or without stenosis) [60], right ventricular dysfunction [61], and increased pulmonary artery pressure are often present in these subjects.

In general, despite the concern about the feasibility of echocardiography in older people related to poor compliance because of cognitive decline and limited visibility of the acoustic window due to underlying pulmonary conditions or concomitant obesity, the use of more recent techniques, such as speckle tracking and deep learning technology, may support physicians in significantly improving management of these patients through early detection of structural and functional cardiac anomalies [60,61,62].

## 3. Concerns and Evidence of Main Treatments

### 3.1. Standard Therapy

Recommendations for treatment of HF patients are primarily based on studies and randomized clinical trials (RCTs) that include younger populations, often male and with reduced ejection fraction [22], although older patients, particularly older women with HFpEF, actually represent the majority of patients with HF in clinical practice [63,64].

Indeed, pharmacological treatment of CHF is usually not stratified by age, rather by levels of ejection fraction (HF with preserved ejection fraction, HFpEF; HF with mid-range ejection fraction, HFmrEF; or HF with reduced ejection fraction, HFrEF) [22]. Until now, this classification has played an important role in guiding HF management even in advanced age [65], even though it is well established that the etiology of CHF may be quite different in older people compared to younger subjects and the overall prognosis is usually poorer in the first subgroup.

Standard treatment for CHF includes the use of disease-modifying medications such as angiotensin-converting enzyme inhibitors (ACE-Is) or angiotensin receptor–neprilysin inhibitors (ARNIs) (or angiotensin II receptor blockers [ARBs] in cases of intolerance to ACE-Is or ARNIs), beta-blockers, and mineralocorticoid receptor antagonists (MRA) as first-line therapy [22]. Additionally, sodium-glucose cotransporter 2 (SGLT2) inhibitors are recommended regardless of diabetes status, along with loop diuretics to alleviate signs and symptoms of congestion while maintaining euvolemia at the lowest effective dose [1].

Here we present current evidence about the efficacy and safety of standard therapies in older HF patients, mainly extrapolated from age-subgroup analyses of large clinical trials.

#### 3.1.1. Angiotensin-Converting Enzyme Inhibitors, Angiotensin Receptor-Neprilysin Inhibitors, and Angiotensin Receptor Blockers

Angiotensin-converting enzyme inhibitors (ACE-Is) and angiotensin receptor blockers (ARBs) both act on the RAAS (renin-angiotensin-aldosterone system), the former preventing conversion of angiotensin I in angiotensin II, the latter by blocking angiotensin II vasoconstrictive activity and its stimulation of aldosterone secretion by the adrenal cortex [66,67]. Angiotensin receptor–neprilysin inhibitors (ARNIs) are made of an ARB and an inhibitor of neprilysin, an enzyme that degrades natriuretic peptides, bradykinin, adrenomedullin, and other vasoactive peptides [68].

Since the 1990s, numerous studies, such as the SOLVD trial and the CONSENSUS trial, have demonstrated the efficacy of angiotensin-converting enzyme inhibitors (ACE-Is) and angiotensin receptor blockers (ARBs) in reducing the risk of hospitalization and mortality in HFrEF patients [69,70]. Regarding older subgroups, in the HOPE study (Heart Outcomes Prevention and Evaluation), ramipril significantly reduced cardiovascular mortality by 26%, all-cause mortality by 16%, stroke by 32%, and heart failure by 23% in high-risk elderly patients (>65 years) with pre-existing cardiovascular disease or diabetes mellitus [71,72]. In addition, for the candesartan, the CHARM study (Candesartan in Heart Failure—Assessment of Mortality and Morbidity) found that elderly patients, despite a higher absolute risk of adverse cardiovascular outcomes, attained a similar relative risk reduction and consequently a greater absolute benefit from candesartan treatment, even though they received a slightly lower daily dose (10–15% lower) compared to the placebo group in the 70–79 and ≥80 age categories, with a similar relative risk of adverse effects across all age groups [73]. Similarly, in the VAL-HeFT study, valsartan was superior to a placebo, significantly reducing the risk of mortality without showing differences in outcomes between patients aged <65 or ≥65 years (47% of the study population), despite a higher risk of side effects [5,74].

On the other side, controversial results have been derived from clinical trials on the role of ACE-Is and ARBs in older patients with HFpEF. In the I-PRESERVED trial (average age of participants 72 years), irbesartan (compared to placebo) did not improve the primary composite outcome (i.e., all-cause mortality or hospitalization for cardiovascular causes such as heart failure, myocardial infarction, unstable angina, arrhythmia, or stroke), nor did it improve secondary outcomes (which included death from heart failure or hospitalization for heart failure, death from any cause, cardiovascular death, and quality of life) [75,76]. Controversial results were also derived from the PEP-CHF study, demonstrating that in patients over 70 years of age with a mean FEVS of 65%, perindopril significantly reduced hospitalizations for HF at one year while improving functional class and exercise capacity [75,77]. On the contrary, Kitzman et al.’s trial showed that treatment with enalapril for 12 months in a population of HF individuals aged 70 years and older (80% women) did not enhance peak exercise capacity, aortic distensibility, or diastolic LV function, although there was a trend towards improved quality of life and modest reductions in resting diastolic blood pressure and peak systolic blood pressure during exercise and a slight improvement in carotid artery distensibility [75,78].

Despite more dated studies reporting better tolerability of losartan compared to captopril in older HF patients (aged ≥65 years), although without superiority in terms of mortality [79,80], current guidelines suggest the use of ARBs as second-choice medications when ACE inhibitors are not sufficiently tolerated [22]. In addition, prescription of the right dosage should be achieved in all age subgroups, based on data that patients aged ≥80 years receiving the target dose of ACEI/ARB have higher survival rates for all-cause mortality at five years compared to those receiving <50% of the target dose (HR = 0.6, 95% CI 0.4–0.9, *p* = 0.033) [81]. On the other hand, the combination therapy of ACE inhibitors and ARBs in older patients has been linked to substantially higher rates of discontinuation, slightly elevated rates of hyperkalemia, and no observed benefits regarding mortality or progression of chronic kidney disease [82].

In the last decade, angiotensin receptor–neprilysin inhibitors (ARNIs) have been recognized as both a reliable replacement for ACE-Is in patients with HFrEF and persistent symptoms despite optimized therapy and a first-line therapy instead of ACE-Is [22,83]. The first study, the PARADIGM-HF, (8399 participants: 2557 subjects aged between 65–75 years; 1563 subjects aged ≥75 years) demonstrated the superiority of the ARNI sacubitril/valsartan compared to enalapril in reducing hospitalizations for HF exacerbation, cardiovascular mortality, and all-cause mortality in patients with HFrEF [22], without a significant increase in the risk of severe angioedema and with a lower discontinuation rate compared to enalapril [84]. The analysis of age subgroups, including those aged ≥80 years (number of patients: 587—7%) and those aged ≥85 years (number of patients: 121—1.44%), corroborated the greater efficacy of enalapril and a favorable benefit-risk profile [85]. The long-term benefits of ARNI were also confirmed in a recent retrospective real-world study by Maury et al., even in patients aged ≥75 years with HFrEF and those classified as NYHA III/IV or NYHA IV [86].

ARNI showed a general good tolerability in the elderly, with similar adverse effects across all age groups, although older patients reached lower doses compared to younger ones after medication titration. Interestingly, discontinuation of the medication occurred more frequently in younger patients, maybe due to a worse prognosis [87], even if the most common side effect of the drugs, which is symptomatic hypotension, tended to be more frequent in the elderly. Data for HFpEF patients were less significant, as reported in the PARAGON-HF study, involving 4822 patients (46% were aged ≥75 years) and showing that sacubitril/valsartan did not statistically significantly reduce the primary endpoint of cardiovascular mortality or hospitalization for heart failure compared to valsartan alone. However, subgroup analyses revealed a significant reduction in hospitalizations among subjects with left ventricular ejection fraction below the median value of 57% and in women, along with a more modest decline in estimated glomerular filtration rate (eGFR) compared to valsartan and a lower risk of composite renal events compared to valsartan [75,88].

As expected, following the FDA approval of ARNIs, the use of sacubitril/valsartan in patients with HFrEF has steadily increased, resulting in a decrease in ACE inhibitor prescriptions. However, several studies indicate that patients with a higher burden of comorbidities and frailty, particularly those with HFrEF aged over 85 years, are less likely to receive the guideline-recommended therapy (GDMT), specifically ARNIs [89]. Even so, regardless of frailty status, patients receiving at least one class of GDMT are associated with lower rates of heart failure hospitalizations and all-cause mortality compared to those not receiving any GDMT for over four years [89,90]. Therefore, an increased awareness and knowledge about the efficacy and safety of new strategies should be encouraged in all subgroups, especially in frailer and more complex subjects.

#### 3.1.2. Mineralocorticoid Receptor Antagonists

Mineralocorticoid receptor antagonists (MRAs) act by blocking aldosterone action in renal tissue, essentially preventing fluid and sodium retention and potassium loss [91].

Despite the established benefit of mineralocorticoid receptor antagonists (MRAs) in reducing morbidity and mortality in patients with HFrEF, they are still under-prescribed, mainly in older patients, likely due to physicians’ concerns about increased risk of renal function deterioration and hyperkalemia [92,93]. An important meta-analysis of the three major trials of CHF in the elderly (≥75 years of age) (RALSE, EMPHASIS-HF, TOPCAT-Americas) highlighted that the use of MRAs reduces the risk of cardiovascular death or first hospitalization for HF uniformly across the three different ejection fraction classes, with more pronounced beneficial effects observed in trials focusing on HFrEF and HFmrEF compared to those on HFpEF [92].

The use of MRAs in the elderly with HFmrEF and HFpEF is less clear due to the ongoing debate surrounding the results of the OPTIMIZE-HF study (mean age 80 years, mean ejection fraction 54%, 59% women), which showed no association between MRA and a decrease in all-cause mortality or hospitalization for heart failure [94], as well as of the TOPCAT study (≥75 years), which showed an insignificant reduction in the primary endpoint of cardiovascular death, hospitalization for heart failure, or cardiac arrest [95].

However, with the recent publication of the FINEARTS-HF study (mean age 72 years) and the meta-analysis by Jhund et al., there is comprehensive evidence that non-steroidal MRAs (finerenone) in HFmrEF or HFpEF patients resulted in a significant lower rate of a composite of total worsening HF events and death from cardiovascular causes than placebo [93,96].

#### 3.1.3. Beta-Blockers

Beta blockers counteract abnormal sympathetic stimulation typical of heart failure by inhibiting cardiac β1 adrenergic receptors; of note, carvedilol, a non-selective beta-blocker, also acts on α-adrenergic receptors, thus determining decreased vascular tone and afterload [97,98]. The goal of beta-blocker therapy in patients with sinus rhythm is to achieve a heart rate between 50 and 70 beats per minute, while this target is not mandatory in patients with reduced or mildly reduced ejection fraction and atrial fibrillation [99].

Several randomized studies and meta-analyses have demonstrated the efficacy of beta-blockers in patients with HFrEF in terms of reducing the risk of sudden cardiac death, cardiovascular death, and all-cause mortality [81,82,83,84,85]. In particular, the absolute risk reduction in mortality was shown to be 5.5% with bisoprolol, 4.6% with carvedilol, 4.5% with metoprolol, and 2.2% with nebivolol [99]. It is likely that the lower reduction in mortality observed with the last drug may be attributed to the older age of patients on nebivolol therapy included in the SENIORS study (mean age of 76 years) [99,100,101,102,103].

In addition, the meta-analysis by Kotecha D et al. confirms that patients with HFrEF in sinus rhythm should receive beta-blockers to reduce the risk of death and hospitalization, regardless of age or sex [104], as well as the study by Cleland et al., who demonstrated that beta-blockers can reduce cardiovascular mortality in patients (average age 65 years) with HFrEF and HFmrEF in sinus rhythm compared to a placebo [105,106]. Notably, beta-blockers were effective in reducing mortality across all age groups; specifically, there was a 35% reduction in mortality risk among patients with a mean age of 68 years and a 23% reduction among those with a mean age of 75 years [104].

While beta-blockers represent a mainstay of treatment for HFrEF, the same results were not confirmed in studies on HFpEF patients [106,107,108], mainly in advanced age. In particular, in the OPTIMIZE-HF study, the initiation of beta-blocker therapy did not improve outcomes in patients aged over 75 years with HF and left ventricular ejection fraction (LVEF) >40% [109]. This result was also confirmed by Patel et al., who found no improvements by beta-blockers in all-cause mortality in older patients (mean age of 81 ± 8) with HFpEF [106,107]. Similarly, carvedilol did not improve mortality or hospitalization for heart failure in elderly patients with HFpEF in the Japanese J-DHF study (aged ≥75 years) [108].

It is worthy of consideration that beta-blockers are not equally tolerated among older patients, necessitating the customization of therapeutic interventions in terms of tolerability and drug titration [22]. Age has been significantly associated with the rate of therapy discontinuation and the failure to reach the necessary therapeutic target [99,110,111]. Therefore, careful selection should always be performed when prescribing these drugs in order to judiciously balance safety and efficacy. In the CIBIS-ELD study, the use of bisoprolol resulted in a greater reduction in heart rate and more dose-limiting bradycardic adverse events [110]; conversely, carvedilol was associated with a reduction in forced expiratory volume and more non-dose-limiting pulmonary adverse events [110].

#### 3.1.4. Sodium-Glucose Co-Transporter 2 Inhibitors

Sodium-glucose cotransporter 2 (SGLT2) inhibitors block the homonymous renal cotransporter, increase urinary glucose excretion determining osmotic diuresis and improving congestion; furthermore, SGLT2 inhibitors reduce arterial stiffness, improve cardiac metabolic efficiency and contractility, prevent cardiac remodeling, and restore proper activity of the sympathetic nervous system [112].

Until the 2023 update of the ESC guidelines, sodium-glucose cotransporter 2 (SGLT2) inhibitors dapagliflozin and empagliflozin were recommended for all patients with HFrEF, regardless of their diabetic status [22]. Since 2023, with the Focused Update of the 2021 ESC Guidelines, SGLT2 inhibitors have entered the first line of treatment for patients with HFmrEF and with HFpEF after demonstrating through the EMPEROR-Preserved and DELIVER studies that they achieve the composite endpoint of cardiovascular death or reduced hospitalization for heart failure [1,113,114].

Currently, randomized clinical trials (EMPEROR-reduced, EMPEROR-preserved, DELIVER, DAPA-HF) show that older patients have good tolerability for SGLT2 inhibitors [115] and experience a similar or even greater relative risk reduction compared to younger individuals regarding cardiovascular mortality or hospitalization for HF [113,114,115,116,117]. Furthermore, SGLT2 inhibitors have demonstrated nephroprotective effects (reducing albuminuria and the decline in glomerular filtration rate) even in the older population at risk for chronic kidney disease (CKD), with no significant differences compared to younger patients (CREDENCE ≥ 65 years: HR 0.77; DAPA-CKD ≥ 65 years: HR 0.58; EMPA-KIDNEY ≥ 70 years: HR 0.65) [118,119,120].

However, the side effects associated with SGLT2 inhibitors, such as volume depletion (especially when used concomitantly with diuretics), lower limb amputations, urinary tract infections, and worsening urinary incontinence, may be more common in the elderly, although their incidence remains relatively low. This is why careful monitoring of renal function, blood pressure, and potassium levels is always advisable in these patients [115,121]. The benefit-risk profile of SGLT2 inhibitors in frail older adults has yet to be fully explored; however, some studies have demonstrated good tolerability and better results in frailest categories. Indeed, patients with a higher degree of frailty (assessed using the Rockwood cumulative deficit approach) showed greater improvement with dapagliflozin compared to those with a lower degree of frailty [122].

Furthermore, although the study documented a higher frequency of adverse reactions and treatment discontinuation in patients with greater frailty, this was not attributed to dapagliflozin [75,122]. Similarly, in a post-hoc analysis of the DAPA-HF study, dapagliflozin was shown to improve all examined cardiovascular outcomes, regardless of frailty status, although absolute reductions were greater in frailest patients [75,123].

#### 3.1.5. Loop Diuretics

Loop diuretic agents exert their natriuretic actions by binding Na^+^, K^+^, 2Cl^−^ cotransporters (NKCC) in a renal Henle loop [124].

Diuretic therapy aims to achieve and maintain a state of euvolemia at the lowest effective dose. Guidelines recommend loop diuretics to reduce signs and/or symptoms of congestion in patients with symptomatic heart failure, placing them at level of evidence I for the treatment of HFmrEF and HFpEF [1,22] and demonstrating a reduction in hospitalization rates [5].

However, there is no evidence from clinical randomized trials suggesting an effect on mortality. Some studies on invasive hemodynamic-guided diuretic therapy (such as the CHAMPION and LAPTOP II trials) support the efficacy of diuretics in reducing congestion in the absence of age-related interactions [75].

In any case, older patients are notoriously more susceptible to adverse reactions due to dehydration, incontinence, reduced renal filtration, hyperkalemia, or hypokalemia. Therefore, close monitoring is mandatory in clinical practice, especially when diuretics are used concomitantly with other therapies that may exacerbate electrolyte imbalance and hypovolemia (such as ACE inhibitors, ARNI, and SGLT2 inhibitors) [5].

A further complication in the management of vascular congestion in older patients regards the observation that diuretic therapy has the most efficacy in the initial phase; however, when administered chronically, it gradually declines. Appropriate patient decongestion, therefore, may not be achieved despite proper dosing and titration of diuretics. This phenomenon is referred to as “diuretic resistance” [125] and may be explained by different mechanisms. Among them, one of the most evident is the counterregulatory effect of neurohormonal activation induced by long-term therapy with these drugs. The reduced sodium sensing at the macula densa level, caused by diuretics, stimulates activation of the renin-angiotensin-aldosterone system [126,127,128]; however, over time, this counterregulatory effect counterbalances the decongestive effect of diuretics, ultimately making these drugs less effective. In addition, renal perfusion may be reduced in the case of low cardiac output, leading to a decrease in blood flow to the kidneys and a reduction in the amount of diuretics that effectively reach the loop of Henle [129]. Several strategies have been proposed to optimize diuretic therapy when diuretic resistance occurs, such as the sequential nephron blockade through the combination of thiazide and thiazide-like diuretics, although this comes at the cost of an increased risk of electrolyte imbalances, especially hypokalemia [130,131]. Other drugs, such as carbonic anhydrase inhibitors [132] or vasopressin antagonists [133], may also be considered, although their use is not explicitly mentioned in current guidelines.

Moreover, the worsening of renal function is not solely caused by a condition of hypoperfusion of the organ but can also result from venous congestion [134], leading to increased renal afterload. These two mechanisms, in addition to reducing the effectiveness of therapy, underlie the so-called “cardiorenal syndrome” [135]. When this vicious cycle of progressive renal function decline sets in, further exacerbating the patient’s HF, the need to rely on ultrafiltration often becomes unavoidable. The ideal timing for initiating this strategy would be when conventional decongestive therapies are no longer sufficient to maintain an appropriate volume status, which is usually when combination diuretic therapy fails to ensure effective adequate volume control. With its efficacy assessed through urinary natriuresis, ultrafiltration should be considered as the next therapeutic step [136,137].

In conclusion, although specific data on treatment of CHF in older subjects are limited and mainly extrapolated from large-scale clinical trials, the standard pharmacological management may be considered well tolerated and generally safe. In any case, a personalized and tailored approach is mandatory, above all in frailer and more comorbid subjects, due to the higher rate of drug interactions, side effects, and therapy discontinuation. Compared to younger people, the assessment of comorbidity status and relative treatments represent a cornerstone in the elderly, both in HFpEF and also in HFmEF and HFrEF, guiding physicians in detecting the safest class and doses among the different available drugs.

### 3.2. Adjunctive Therapies

#### 3.2.1. Ivabradine

Ivabradine decreases heart rate, inhibiting I_f_ channels in the sinus node [22]. The current guidelines recommend ivabradine therapy, classified as having IIa evidence, to reduce the risk of hospitalization for heart failure and cardiovascular mortality in symptomatic patients with an ejection fraction (EF) ≤35%, in sinus rhythm, and with a resting heart rate ≥70 beats per minute despite optimized treatment or if beta-blocker therapy is contraindicated or intolerable [22].

There is limited scientific evidence regarding the role of ivabradine in older HF patients. The SHIFT study (Systolic Heart Failure Treatment with the If Inhibitor Ivabradine Trial) demonstrated a reduction in the composite endpoint of cardiovascular mortality and hospitalization for heart failure, irrespective of age, with no significant differences in adverse effects. However, the studied population (of 6492 participants) was relatively young, with a mean age of 60.4 ± 11.4 years, and patients aged between 60 and 69 and those aged ≥69 constituted 27% and 26%, respectively [5,138]. It is interesting to note, however, that in a subgroup of 722 very old patients, aged ≥75 years (47% of the 1712 patients in the >69 years group), the composite endpoints occurred less frequently with ivabradine compared to a placebo, although the intergroup differences were not significant [138].

Regarding the tolerability and safety of the drug, the RELIF-CHF and SIGNIFY studies (with a mean age of participants at 65 years) identified arrhythmias, symptomatic bradycardia, atrial fibrillation, nausea, and visual disturbances (phosphenes) as the main side effects, thus necessitating careful monitoring of this therapy in older subjects [139,140].

#### 3.2.2. Digoxin

Digoxin is a positive inotropic agent, whose action consists of inhibition of Na^+^-K^+^-ATPase and secondary activation of the Na^+^-Ca^2+^ membrane exchange pump on cardiomyocytes, which drives intracellular flux of calcium; furthermore, digoxin exerts vagomimetic effects on the atrioventricular (AV) node, stimulating the parasympathetic nervous system [141].

Digoxin may be considered for the treatment of HFrEF in sinus rhythm regardless of therapy with ACE inhibitors (or ARNIs), beta-blockers, and mineralocorticoid receptor antagonists (MRAs) [22] or for the treatment of symptomatic patients with HFrEF and concomitant atrial fibrillation with high ventricular rate when other therapeutic options cannot be pursued (class of evidence IIb) [22].

The DIG trial (7788 patients; 2885 aged 60–69 years; 2092 aged 70–79 years) demonstrated that digoxin reduces all-cause hospitalizations, HF hospitalizations, and death or hospitalization due to heart failure regardless of age [142,143], with an intermediate effect on HFmrEF and little effect on HFpEF compared to HFrEF [106,144]. However, the study recommends caution in the use of digoxin in the elderly due to its narrow therapeutic window combined with renal dysfunction, particularly in old women with low body mass index [5,145].

The most common effects of digoxin toxicity were ventricular fibrillation or tachycardia, supraventricular arrhythmia, and second- or third-degree atrioventricular block, although the increased risk of such toxicity was similar across all subgroups in the DIG study [142].

In summary, as also suggested by the ESC guidelines, digoxin should be administered with caution in female old and frail individuals, those with hypokalemia, or those who are malnourished [22].

#### 3.2.3. Vericiguat

Vericiguat is a soluble guanylate cyclase stimulator that enhances the activity of cyclic guanosine monophosphate (cGMP), which is involved in the regulation of protective cardiovascular, kidney, and metabolic actions [146].

Vericiguat represents an innovative therapeutic option for patients with HFrEF on optimized medical therapy [68], mainly for those who have experienced recent exacerbations and are at high risk of hospitalization [22].

The results of the VICTORIA study demonstrated that vericiguat reduces cardiovascular mortality and hospitalizations due to heart failure, although the benefits vary among different patient subgroups [147]. Specifically, no significant reduction in the primary endpoint was observed in the age groups ≥65 years (RR: 0.94; 95% CI: 0.84–1.04) and ≥75 years (RR: 1.04; 95% CI: 0.88–1.23) compared to the placebo group [148]. However, in a very common clinical context characterized by worsening renal function in heart failure patients and secondary hyperkalemia, easily leading to the discontinuation of ARNI/ACE inhibitor/SGLT2 therapies [148], vericiguat may represent a valuable option for clinicians, as it can be safely used in patients with severe renal impairment (eGFR ≥15 mL/min). However, as of now, evidence is still limited, and further studies may be necessary to fully clarify its efficacy and safety profile in older patients, mainly considering the common effects of hypotension and syncope.

On the other side, studies such as SOCRATES-PRESERVED and VITALITY-HFpEF have not demonstrated significant efficacy of vericiguat subjects with HFpEF, although the drug has been well tolerated [148,149,150,151].

#### 3.2.4. Management of Anemia

Anemia is a condition present in many elderly individuals and is caused by multiple factors, including chronic inflammation, renal insufficiency, and iron deficiency. The latter, defined as a serum ferritin concentration <100 ng/mL or 100–299 ng/mL with a transferrin saturation (TSAT) <20% [152], can impair functional capacity, precipitate circulatory decompensation, and promote musculoskeletal dysfunction and is associated with frailty, independently of anemia [153]. Recent studies and meta-analyses have demonstrated that in patients with HFmrEF and HFrEF, intravenous iron supplementation, particularly with ferric carboxymaltose, improves symptoms (with an increase in NYHA class and exercise capacity) and quality of life [154] and reduces hospitalization rates [155,156,157], without, however, providing benefits related to reducing cardiovascular and all-cause mortality [158]. For this reason, intravenous supplementation with ferric carboxymaltose is currently recommended by international ESC guidelines [1,22].

As of now, there are no evidence-based recommendations for the management of anemia in patients with cardiorenal syndrome (CRS), although treatment of this condition should be multifactorial, as it must simultaneously address HF, CKD progression, and complications, such as anemia itself [159,160]. The PIVOTAL study (number of participants: 2141, mean age: 64 years [52,53,54,55,56,57,58,59,60,61,62,63,64,65,66,67,68,69,70,71,72,73,74,75]) demonstrated that in patients with end-stage renal disease undergoing hemodialysis, high-dose intravenous iron administration reduces HF events (first event and recurrent events) [161]. Some studies have evaluated the efficacy of combined therapy with iron and erythropoietin in patients with CRS, showing that such supplementation can improve myocardial functional capacity, left ventricular (LV) function, LV remodeling, NYHA class, BNP levels, and overall quality of life [161,162,163]. Finally, in patients with CRS, long-term treatment with erythropoietin (52 weeks) significantly increases Hb levels and reticulocyte count and improves symptoms but does not have a clear impact on reducing hospitalization rates in this patient population [163]. However, according to Vullaganti et al., in elderly patients with HFpEF (mean age: 77 ± 11 years), the use of epoetin alfa or oral iron supplements does not lead to improvements in cardiac structure, exercise capacity, or quality of life compared to a placebo [164].

Further research is needed to optimize strategies for managing anemia in patients with CRS.

### 3.3. Cardiac Resynchronization Therapy and Implantable Cardioverter-Defibrillator

Cardiac resynchronization therapy (CRT) is recommended to improve symptoms and reduce morbidity and mortality in symptomatic HF patients in sinus rhythm who have a QRS duration ≥150 ms with left bundle branch block morphology and an ejection fraction (EF) ≤35% despite optimized therapy [22] or in patients with HFrEF who have an indication for ventricular pacing due to high-degree atrioventricular (AV) block [22]. On the other side, the implantable cardioverter-defibrillator (ICD) is recommended both for secondary and primary prevention to reduce the risk of sudden cardiac death and all-cause mortality in selected patients [22].

CRT has been shown to improve HF symptoms and reduce mortality. However, there are limited data specifically addressing the effects of CRT in individuals over 65 years of age [5], except for the randomized clinical trials MIRACLE and MIRACLE-ICD, showing significant improvements in NYHA class and left ventricular ejection fraction in all age subgroups (<65, 65–75, and >75 years) [165].

Based on the concept that left ventricular telediastolic diameter remodeling and reduction in QRS amplitude were significantly more pronounced in the elderly [166], similar positive results were later confirmed by the MADIT-CRT study and by Behon A et al., showing that CRT-D was associated with a significant clinical benefit in patients aged ≥60 years and those aged ≥75 years, without any increase in device-related adverse events [167,168].

Similarly, one study reported that ICD implantation may be beneficial for older patients (≥75 years), with no differences in complications related to the ICD (operative, hospital-related, or long-term) between older and younger patients [169]. However, the benefits of ICDs, especially for primary prevention, are less consistent in older patients compared to younger patients, even resulting in controversial results in those patients with non-ischemic heart failure [5].

Therefore, despite positive findings, it remains unclear whether older patients actually experience improvements with an ICD or cardiac resynchronization therapy (CRT) compared to younger patients, as death may be linked to a series of causes different from the cardiovascular disease itself. For instance, the Italian Clinical Service Project showed that 22.6% of mortality among older participants (of 6311 study participants) was attributable to comorbidities [170]. Consequently, potential candidates for CRT and ICD therapy should be carefully selected based on quality of life and survival expectations to better formulate and analyze ethical and cost-effectiveness aspects [5,170].

### 3.4. Uncommon Therapies

Hydralazine is an arteriole vasodilator; its role is to inhibit inositol trisphosphate (IP3)-induced release of calcium from smooth muscle cells’ sarcoplasmic reticulum, preventing myosin phosphorylation [171]. On the other hand, isosorbide dinitrate is a venodilator and vasodilator agent: it determines nitric oxide (NO) release from the endothelium, which causes guanylate cyclase activation; this results in guanosine 3′5′ monophosphate (cGMP) increase, finally determining dephosphorylation of myosin light chains and smooth muscle relaxation [172].

The combination of hydralazine and isosorbide dinitrate may be considered to reduce the risk of death in symptomatic HFrEF patients who are intolerant (or have contraindications) to both ACE inhibitors/ARBs and ARNIs [22]. However, this recommendation is based on the results of a relatively small study, the Veterans Administration Cooperative Study, which included only male patients with symptomatic HFrEF being treated with digoxin and diuretics [22]. There is even less evidence regarding the use of the hydralazine-isosorbide combination in older patients.

A left ventricular assist device (LVAD) may be used to prolong survival and improve quality of life or to sustain the patient until transplantation or resolve contraindications to transplantation [22]. Considering that comorbidities and age are factors that may exclude a patient from transplantation, it is not surprising that there is limited literature supporting the implantation of an LVAD in older patients. Some studies involving small populations have not shown significant differences in survival rates among patients with heart failure aged 65 to 72 years without renal dysfunction compared to younger patients, although there has consistently been an increased rate of LVAD-related complications (such as sepsis and gastrointestinal bleeding) [5,173,174]. On the other hand, the INTERMACS registry reported a significant decline in 2-year survival among LVAD patients over 70 years of age (71% vs. 63%), and age itself was found to be an independent predictor of mortality [175]. Table 3 summarizes the main take-home messages relative to different pharmacological options for CHF in the elderly.

### 3.5. In-Hospital Pharmacological Treatment

Managing ADHF in the older population can be particularly challenging, even with conventional therapies, due to the high rate of multimorbidity and chronic polytherapy and consequent increased risks of potential side effects and drug interactions [176].

Diuretics represent the cornerstone treatments for ADHF, since these patients often exhibit symptoms related to congestion and volume overload. Current guidelines recommend initiating intravenous diuretics with low doses, gradually increasing based on the patient’s clinical response. If the patient is already on diuretic therapy at home, it is still advisable to start with a diuretic dose that is one to two times higher than the patient’s usual daily dose [22].

Although guidelines do not provide specific recommendations on diuretic use in the elderly, clinical practice commonly involves their administration alongside close monitoring of volemic status and urine output. Nevertheless, intravenous diuretics must be used carefully since older patients often have a higher risk of hypovolemia and volume depletion [177]. Notably, diuretic use during hospitalization for AHF is higher in older than in younger patients [16], possibly explained by the fact that the first group of patients experience a greater reduction in renal filtration, as previously mentioned, and therefore require higher doses of the drug to achieve good efficacy. However, the progressive decline in renal function with advancing age represents a concern for diuretic titration, making the identification of the most suitable dose more difficult [178].

It is known that in patients with a systolic blood pressure >110 mmHg, intravenous vasodilators can be used, as they are effective in reducing both afterload and venous return [22]. However, some studies suggest that in older patients, the use of nitrates in the emergency department does not lead to a significant reduction in either mortality or hospital stay [179], nor does early-stage nitrate bolus administration affect 30-day mortality when compared to standard therapy [180]. In general, therefore, vasodilators should be considered in patients with hypertension, though with caution in elderly subjects, and should be avoided in those subjects with higher risk of hypoperfusion and hypovolemia.

There are limited data on the efficacy and/or adverse effects of more intensive drugs, such as vasopressors, in the geriatric population. In general, inotropic agents tend to be used less frequently in patients older than 80 years [14,16]. Very few data are available on dobutamine, which appears to exert a reduced effect on improving stroke volume and cardiac output in comparison with younger patients, and increasing the dose does not improve these outcomes [181]. It is likely that this finding can be attributed both to a global diminished beta-adrenergic responsiveness with advancing age [182] and to vascular aging mechanisms, since older patients show a greater reduction in preload, leading to a smaller increase in terms of stroke volume, according to the Frank–Starling mechanism [181].

In conclusion, lacking dedicated guidelines, ADHF management in older people is mainly based on intravenous diuretics, with limitations on dose titration due to high risk of worsening kidney function, electrolytes imbalance, and hypovolemia. Vasodilators and vasopressors, while useful in specific situations, may be burdened by relevant side effects without showing a significant benefit.

### 3.6. Therapy at Discharge

Optimizing therapy for older patients at discharge according to GDMT can be very difficult. Arrigo et al. performed a sub-analysis of patients aged 65–85 years in the STRONG-HF trial, a multinational, open-label randomized study assessing whether rapid up-titration of medications before discharge would improve quality of life, mortality, and 180-day readmission rates. GDMT included beta-blockers; ACE inhibitors or ARBs (if ACEi-intolerant) or ARNIs; and MRAs. Results showed that this swift GDMT titration is feasible in older patients, entails no substantial risks, and significantly reduces mortality and readmissions in both younger and older populations. However, quality-of-life improvements, a key relevant aspect in this class of patients, were more modest [183].

Similar benefits in terms of mortality reduction were also reported by Sung et al. [18]. In their observational study on 1297 patients (mean age 85.1 years) admitted between 2003 and 2012, they showed that adherence to GDMT with prescription at discharge, both for HFrEF and HFpEF, positively influenced mortality at 1 and 3 years, especially among patients with HFrEF. However, the study only evaluated whether medications were prescribed at discharge, without assessing dosages or adherence during follow-up, and it did not account for new drugs such as SGLT2 inhibitors and ARNIs. On the other side, Akita et al. [184], in a multicenter retrospective study using data from the WET-HF registry (2005–2014), confirmed that very old subjects (>80 years) were less likely to receive GMBT prescription at discharge compared to younger patients (<80 years) but also showed that GMBT was associated with reduced mortality and improved prognosis only in the cohort younger than 80 years [184].

Data about ARNIs and SGLT2 in the long term are still being updated. Subgroup analyses of the PARADIGM-HF study indicate that ARNIs reduce mortality and readmissions across all ages [185]. As in the PIONEER-HF study, ARNI use was considered feasible even in patients over 65 years of age with HFrEF [186]. However, major concerns derived from a long-term strategy, since the main predictor of successful dose escalation was just identified in younger (<65 years) ages. It is likely that the risks of hypotension, worsening renal function, and hyperkalemia may significantly impact clinicians’ decisions about the dosage to prescribe while sacrificing the benefits of higher doses.

Regarding the effects of SGLT2 inhibitors, data on very old and severely frail individuals are limited, since they were often excluded from relative studies. Nonetheless, empagliflozin use was assessed in patients older than 70, demonstrating both benefits and a favorable safety profile, even when initiated early in hospitalization [187]. Further studies may clarify the long-term benefits in all age subgroups.

In conclusion, in elderly patients, GDMT does not appear to be unfeasible; however, overall, its overall effectiveness and long-term benefits seem to be diminished compared to in younger patients. The reasons underlying these findings are complex and include first and foremost the presence of multiple comorbidities, which can either contraindicate or increase the risk associated with certain therapies. Furthermore, the pharmacological response in elderly patients may be altered due to age-related changes in cardiovascular physiology and drug interactions [188].

## 4. Typical Prognostic Factors

Up until now, different studies have analyzed a series of predictors of severe adverse events, hospitalization, and mortality within the short term (<30 days) and long term for patients admitted for ADHF [14,19]. Despite the heterogeneity in the timing of adverse event assessments and in cohort selection, almost all studies show that age, frailty, and comorbidity status represent the most significant factors influencing prognosis, rather than the cause of the acute exacerbation of HF itself [14]. Frailty, in particular, has been directly linked to a higher risk of rehospitalization after discharge and to the number of readmissions within six months [189]. Indeed, a low Barthel Index (<60) and a high Charlson Comorbidity Index (>2), assessed at the time of hospitalization for acute heart failure (AHF), were identified as independent predictors of 1-year mortality in older patients [190]. Table 4 summarizes the most relevant results on this topic.

It is worth noting that in general, prognostic stratification of patients admitted for ADHF is based on different parameters according to age. Regardless of whether, in fact, reduced ejection fraction, elevated troponin levels, and AHF etiology play a more significant role in younger subjects, on the other side, frailty and comorbidities dominate in influencing adverse outcomes and mortality in the elderly.

Prognostic score have also been proposed to specifically evaluate the risk of in-hospital mortality in old patients admitted for ADHF [191,192]. Jia et al. proposed a predictive mortality score (RPSS, Risk Prediction Score System), for patients aged over 60 years, including heart rate, left ventricular ejection fraction, pH value, renal dysfunction, and NT-proBNP, resulting in a reliable predictive model correlated with patient mortality [191]. In addition, Gok et al. proposed a model for patients over 65 years of age, based on the following variables: age > 77.5 years, anemia, a history of hemodialysis, use of inotropic drugs, ICU stay longer than 6.5 days, and uncontrolled hypertension. A significant relationship was observed between higher scores and mortality rates [192]. Therefore, it seems clear that pre-existing kidney dysfunction and related fluids and electrolytes imbalance play a relevant prognostic role in the short term, as confirmed in the other studies reported in Table 4.

Regarding the “very old” population (>85 years), mainly represented by women with HFpEF and fewer traditional cardiac comorbidities (such as hypertension, ischemic heart disease, and diabetes) compared to younger patients, in-hospital mortality, one-year all-cause mortality rates, and rehospitalization risks were significantly increased [16,21,193,194,195]. Atrial fibrillation has been described as a key negative prognostic factor [21,193], and infections result in the most prevalent causes of death [194]. Even so, patients over 90 years face a higher burden of comorbidities and are usually characterized by a poor functional status, severe cognitive decline, higher NYHA classes, and renal insufficiency, all of them representing key predictors of a worse outcome [196], together with oxygen saturation <90% and hyponatremia [195].

## 5. Transthyretin Amyloid Cardiomyopathy

Wild-type transthyretin cardiac amyloidosis (wtTTR-CA) represents an underdiagnosed cause of HFpEF typical of advanced age (the median age at diagnosis is >70 years) [197], as up to 16% of patients over 65 years old with cryptogenic left ventricular hypertrophy (LVH) or HFpEF or severe aortic stenosis may have wtTTR-CA [22]. Cardiac amyloidosis is a multisystemic disease with a wide range of symptoms: neurological and cognitive, gastrointestinal, musculoskeletal, ophthalmological, and autonomic dysfunctions, mimicking or exacerbating other age-related disorders, making recognition particularly challenging in the early stages of the disease [198]. However, diastolic heart failure with preserved ejection fraction remains the most common clinical presentation in patients with ATTR-CA [199]. The diagnosis of ATTR-CA should be suspected in patients aged 65 years or older with unexplained left ventricular thickness ≥12 mm on echocardiography and at least one of the “red flags” [200] reported in Table 5.

It is estimated that approximately 16% of patients referred for transcatheter aortic valve replacement (TAVR) are affected by ATTR-CA [201]. Recently, the RAISE score (based on remodeling, age, injury, and systemic and electrolyte imbalance) has been developed to assess the probability of concomitant ATTR-CA in patients with aortic stenosis. In consideration of the benefits reported by TAVR in very old patients compared to higher risks of a surgical aortic valve replacement, this score may represent a reliable tool alongside frailty assessments to better characterize those patients who are candidates for this intervention [202].

Non-invasive diagnostic tools can be considered only for ATTR-CA [200], first of all represented by the technetium nuclear scintigraphy with bone-seeking radiotracers [197,198]. Moreover, besides basic echocardiography, other techniques may be used to support the differential diagnosis between ATTR-CA and HFpEF, such as the speckle-tracking echocardiography, able to visualize apical sparing, and cardiac magnetic resonance imaging (MRI), showing diffuse subendocardial or transmural late gadolinium enhancement [197,198,203].

It is known that ATTR-CA infiltrates the atria and is characterized by biatrial enlargement, favoring the onset of electrophysiological complications, such as conduction disorders and atrial fibrillation, typical features themselves of advanced age [198,204]. In any case, the multi-organ clinical manifestations of ATTR, mainly if associated with age-related physical decline, expose most of the patients to the risk of worsening functional capacity with progressive impairment of mobility and associated complications, including falls [198].

There is currently no evidence supporting the use of standard HF therapies, often poorly tolerated, except for diuretics [200]. Tafamidis is the only approved disease-modifying drug, an oral TTR stabilizer that reduces its dissociation [205]. Two other agents, inotersen and patisiran, are approved for hereditary transthyretin amyloid polyneuropathy (ATTRh) and reduce TTR production in the liver [206,207]. Tafamidis has shown significant benefits, especially in patients with early-stage disease (NYHA class I-II), improving survival and reducing hospitalizations and symptoms [205]. However, due to its high cost and the lack of long-term data, a thorough geriatric assessment by a multidisciplinary team is crucial to identify old patients who would truly benefit. In addition, the use of anticoagulant therapy and pacemaker implantation for high-degree atrioventricular blocks and/or symptomatic bradycardia in old patients with ATTR-CA are common and associated with a worse prognosis [204].

Therefore, an interplay with palliative care specialists should be encouraged, mainly in the frailest patients, in order to develop the best personalized care plans for long-term management [198,208].

## 6. Palliative Care

The World Health Organization (WHO) defines palliative care (PC) as an approach facing life-threatening illnesses through physical symptoms prevention and relief and treatment of psychosocial and spiritual problems, with the aim to improve patients and their families’ quality of life (QoL) [209].

According to WHO, adults affected by cardiovascular diseases represent about one-third of patients with chronic diseases in need of palliative care [209]. Despite significant therapeutic advances over the decades, in fact, the prognosis for HF remains poor, with a five-year mortality rate of up to 50% [210,211]. Many randomized controlled trials have recently underlined the role of PC in HF. In 2014, Sidebottom demonstrated that PC could improve acute HF patients’ QoL, symptom burden, and depression [212]. Later, the Palliative Care in Heart Failure (PAL-HF) trial showed that PC provided consistently greater benefits in QOL, depression, anxiety, and spiritual well-being compared to usual care in advanced HF patients [213]. Similar results were obtained from the Collaborative Care to Alleviate Symptoms and Adjust to Illness (CASA) trial [214], showing both fatigue and depression reduction from PC intervention, and by Bakitas et al., reporting pain improvement from early PC in HF patients [215].

However, the actual integration of PC in the management of HF is still quite nebulous. As an example, Greener [216] highlighted that only 6% of hospitalized HF patients at high risk of mortality received PC services; similarly, a few years later, the Mandawat’s cross-sectional analysis assessed a 7.6% PC use for 4474 American veterans affected by severe HF [217]. Interestingly, these absolute PC rates are similar to the ones in chronic obstructive pulmonary disease (COPD) but lower than those from PC referral in oncology (5.1% and 17%, respectively) [218,219].

It seems clear, therefore, that PC in HF is formally recognized but still underutilized. It is likely that the main concern for physicians involves the right timing to propose PC management in such a chronic condition. In particular, the AHA/ACC/HFSA guidelines specify that a PC approach starts from every member of the patient’s primary care team (primary care physicians, cardiologists, other specialists, nurses, psychologists, and so on), targeted to satisfy a patient’s basic palliative needs, to communicate disease prognosis, to clarify treatment goals, and to define a patient’s treatment preferences [68].

This first phase of PC can be referred to as “primary palliative care” [68]. However, there are no specific criteria identifying a “true early period” to start PC [220], and particularly to start primary care PC. Several clinical tools have been suggested to define a clinical status worthy of PC, mainly consisting of general indicators for decline (weight loss, functional status, recent hospitalizations, surprise questions, and so on), but none of these have been validated or widely implemented in HF clinical practice [221].

When a patient’s needs become more challenging as a consequence of HF progression, a palliative care specialist should be consulted, initiating the so-called “secondary palliative care” [68]. In this context as well, the right timing to introduce this approach is not standardized. However, American guidelines suggest the introduction of a PC specialist for multimorbid, frail, or cognitively impaired patients, advanced HF stages with symptomatic burden despite optimal medical therapy, and patients in need of device implantation or inotropic support or temporary mechanical circulatory support (MCS), with an expected survival time <6 months) [68]. Meanwhile, the ESC 2020 position paper [222] highlights a series of possible scenarios for the involvement of a PC specialist in HF patient treatment, detailed in Table 6 [222]. Similarly, Slavin et al. suggested that advanced NYHA (III-IV), frequent hospitalizations (≥2 in the last year), or indication to implant ICD or CRT could be possible key criteria to consider PC intervention [220].

In both primary and secondary palliative care, there is a wide and growing range of HF symptoms that clinicians should give attention to as the disease progresses. The most common ones and their relative management are as follows:

- Dyspnea and breathlessness: optimal HF medical therapy aside, opioid (morphine) use may be considered; opioids also act on anxiety, as do benzodiazepines, which traditionally represent a second–third-line treatment [222,223,224].
- Pain: NSAIDs are generally avoided in order to not worsen renal function [224]; anticonvulsants, antidepressants, and opioids (tramadol, oxycodone, hydromorphone, fentanyl) are preferred [223].
- Anxiety, depression: benzodiazepines are first choice for anxiety; selective serotonin reuptake inhibitors (SSRIs) are indicated for depression, although their efficacy has limited evidence [222,225,226].

Finally, PC in HF patients also involves cardiac device management. Patients with pacemakers may benefit from the device through all stages of PC [227]. Deactivating the resynchronization component in CRT-Ps (cardiac resynchronization therapy pacemakers) should be avoided since this could worsen HF-related symptoms (breathlessness, asthenia, and so on). Instead, deactivation in defibrillator devices can be considered in patients at end of life for preventing frequent, painful, and useless device shocks [227,228,229].

Limited data are available with regard to LVADs in this setting. There is no agreement on this topic, so potentially life-sustaining therapies should be anticipated and discussed at the time of initiation and reconsidered serially with changing medical realities and evolving goals of care [230,231,232,233,234].

## 7. Conclusions

In conclusion, clinical management of HF represents a significant challenge in older patients, as it is influenced by multiple factors, including comorbidities, frailty, and the specific age-related characteristics of this population. With the global aging of the population, the prevalence of HF is rapidly increasing, mainly in women and in individuals with multiple concomitant conditions. The rising number of HF diagnoses highlights the urgent need for a deeper understanding of its pathophysiology, clinical presentation, and successful strategies. Particular attention should be given to elderly patients with left ventricular hypertrophy to rule out potential concomitant red flags, which are crucial to avoid failing to diagnose the increasingly prevalent transthyretin wild-type cardiac amyloidosis.

Given the nature of HF in the elderly, a personalized, patient-centered approach to treatment is essential. Therapeutic regimens must be carefully adjusted to account for the severity of HF, the presence of comorbidities, and the patient’s overall health status. The interest in and attention toward the elderly in the field of cardiology are growing, reflecting the progressive increase in scientific studies that are more inclusive of the older population. Nowadays, we have an increasing number of promising pharmacological options available, such as SGLT2 inhibitors, which may assist clinicians in managing complex and frail patients with HF.

In addition to pharmacological interventions, palliative care has emerged as a key strategy in improving the quality of life for elderly and frail patients with HF. Exploring integrated care models that combine medical, palliative, and psychosocial support is crucial for improving outcomes in the elderly. Effective collaboration across cardiology, geriatrics, and palliative care should be encouraged and will be essential to elaborate comprehensive care plans addressing the full range of challenges faced by elderly HF patients.

## 8. Future Directions

With the increase in life expectancy and the growing prevalence of heart failure among elderly individuals with multiple comorbidities, it has become increasingly urgent to adopt innovative strategies to reduce hospitalizations and improve the quality of life for patients. In this context, it is essential to identify the most vulnerable individuals early, promptly recognize risk factors and precipitants, and intervene effectively to prevent acute events that often lead to repeated hospital admissions. Among the emerging therapeutic options, glucagon-like peptide-1 receptor agonists (GLP-1RAs) have shown potential in reducing adverse events in patients with type 2 diabetes [235] and obesity with preserved ejection fraction [236]. However, their role in the various subtypes of heart failure remains unclear and requires further study [237].

To effectively address this complex condition, prevention and integrated patient management must become priorities. It is necessary to build care networks [238] involving general practitioners, cardiologists, geriatricians, nurses, physiotherapists, and other specialists working synergistically to ensure comprehensive, coordinated, and personalized care. However, in many settings, maintaining effective continuity of care proves challenging due to organizational limitations and available resources. For this reason, strategies such as patient and caregiver empowerment are becoming crucial [239]. Educating patients about disease management and early recognition of clinical deterioration signs is essential for promoting informed and conscious decisions that can improve quality of life.

In recent years, there has been increasing interest in managing acutely decompensated heart failure with outpatient-based care in order to find alternatives to hospitalization and avoid its related complications, especially by delivering intravenous diuretic therapy at home, in community settings, or in hospital day-care units. A recent meta-analysis investigating the safety and effectiveness of outpatient-based management (OPM) compared to the standard inpatient treatment for ADHF shows promising results in terms of 30-day mortality and hospitalization, although a relevant heterogeneity of the included studies was observed [137]. Thus, large prospective multicenter RCTs are required to better establish the safety, clinical, and cost effectiveness of the OPM, mainly in the older subgroup of HF patients.

At the same time, the use of telemedicine and remote monitoring represents an important opportunity to optimize heart failure management. Interventions such as remote monitoring of vital signs—including body weight, oxygen saturation, and respiratory rate—along with electronic transfer of physiological data through advanced devices have been shown to reduce hospitalization rates [240,241] and reduce all-cause and cardiovascular mortality in heart failure patients [241]. Innovative devices like the CardioMEMS HF system or other implantable and wearable monitors also allow for real-time hemodynamic monitoring, enabling timely and personalized therapeutic interventions that can prevent inappropriate hospitalizations [242].

These technological approaches not only have the potential to improve clinical effectiveness but also foster greater collaboration between patients and healthcare providers. They create integrated networks between community services and hospitals [238], allowing for constant and timely contact with patients, enhancing treatment adherence [243], and contributing to improved quality of life and life expectancy. Furthermore, such strategies can help reduce healthcare costs associated with managing chronic heart failure, making the system more sustainable in the long term.

## Figures and Tables

**Table 1:** Differences in clinical presentation of heart failure between young and old individuals.

Young Individuals	Old Individuals
Typical signs and symptoms	Atypical signs and symptoms
Dyspnea	Confusion
Orthopnea	Cachexia
Paroxysmal dyspnea	Somnolence
Fatigue	Anorexia
Lower limb edema	Cough
Reduced exercise tolerance	Asthenia

**Table 2:** Different cut-off values of NT-proBNP proposed according to age and comorbidities.

Studies—Authors	Year	Age and Comorbidities	Cut-Off (pg/mL)
PRIDE—Januzzi JL et al. [43]	2005	<50 years ≥50 years	450 900
ICON-RELOADED—Gaggin HK et al. [44]	2017	<50 years Age between 50 and 75 years ≥75 years	450 900 1.800
Berthelot E et al. [46]	2024	75-85 years >85 years Obesity Male gender Atrial Fibrillation eGFR < 30 mL/min	1.680 2.235 1.375 1.800 2.332 3.474

eGFR: estimated glomerular filtration rate.

**Table 3:** Pharmacological options for chronic heart failure in older subjects.

Drugs	Take Home Messages
ACE-I and ARBs	Efficacy and tolerability like younger patients with HFrEF with quite higher risk of side effects Controversial results in HFpEF ACE/ARB association is not suggested
ARNIs	Efficacy and good tolerability in older patients with HFrEF increased risk of hypotension Less significant data in HFpEF patients
MRAs	Established benefit in elderly with HFrEF (increased risk of renal function deterioration and hyperkalemia Finerenone seems more efficacious in HFmrEF and HFpEF (mainly in renal failure and hyperkalemia)
Beta-Blockers	Efficacy and good tolerability in older patients with HFrEF (mainly bisoprolol) Less significant data in HFpEF patients
SGLT2i	Efficacy and tolerability like younger patients
Diuretics	Need close monitoring especially in association with other therapies that may exacerbate electrolyte imbalance and hypovolemia
Ivabradine	Limited evidence in elderly
Digoxin	Caution in old and frail individuals (mainly female), hypokalemia or malnutrition
Verciguat	Limited evidence (possible use in patients with worsening renal function and hyperkalemia; need close blood pressure monitoring)
Hydralazine/isosorbide	Limited evidence in elderly

ACE-Is: angiotensin-converting enzyme inhibitors; ARBs: angiotensin II receptor blockers; ARNIs: angiotensin receptor–neprilysin inhibitors (ARNIs) (or [ARBs]; HFrEF: heart failure with reduced ejection fraction; HFpEF: heart failure with preserved ejection fraction.

**Table 4:** Prognostic factors of a worse outcome in the elderly.

Authors Year	No. Pts	Mean Age	Factors Influencing Overall Prognosis	Prognostic Factors in the Short Term	Prognostic Factors in the Long Term
Metra M et al. 2015 [20]	2033	72	Age Frailty	*30 days after admission* high BNP NYHA class III-IV low SBP at admission renal dysfunction at admission	*180 days after admission* valvular diseases low serum albumin persistent BNP elevation sustained renal dysfunction
Claret PG et al. 2016 [19]	1658	77.1	Age	*30 days after admission* antiarrhythmic therapy ACS at admission pleural effusion BUN > 12 mmol/L at admission	
Ide T et al. 2021 [13]	13,238	78	Age Chronic comorbidities (e.g., CKD) NYHA class III-IV Aortic stenosis Low albumin levels	*In-hospital mortality* Stage IV-V of CKD Stroke Elevated BNP levels	*4-year follow-up* Prior HF hospitalization Ischemic heart disease Arrhythmias (e.g., VF, VT) Use of CRT-D Diuretic use at discharge Transfer to hospital/nursing facility at discharge

CKD: chronic kidney renal disease; NYHA: New York Heart Association; ACS: acute coronary syndrome; BUN: blood urea nitrogen; BNP: B-type natriuretic peptide; SPB: systolic blood pressure; HF: heart failure; VF: ventricular fibrillation; VT: ventricular tachycardia; CRT-D: cardiac resynchronization therapy-defibrillator.

**Table 5:** Diagnosis of transthyretin amyloid cardiomyopathy.

Essential Finding	“Red Flags”
Left ventricular wall thickness ≥12 mm + one or more of «red flags»	Heart failure ≥65 years Aortic stenosis in ≥65 years Hypotension or normotensive if previously hypertensive Sensory involvement, autonomic dysfunction Peripheral polyneuropathy Proteinuria Skin bruising Ruptured biceps tendon Bilateral carpal tunnel syndrome Subendocardial/transmural LGE or increased ECV on magnetic resonance Reduced longitudinal strain with apical sparing Decreased QRS voltage to mass ratio Pseudo Q waves on ECG Atrio-ventricular conduction disturbance Possible family history of transthyretin amyloid cardiomiopathy Chronically increased troponin levels Known multiple myeloma or MGUS

LGE: late gadolinium enhancement; ECV: extracellular volume; ECG: electrocardiogram; MGUS: monoclonal gammopathy of indetermined significance.

**Table 6:** Main scenarios demanding the integration of specialist palliative care in HF management.

	Possible Scenarios
Useful palliative care Specialist involvement	Refractory or complex symptoms Spiritual or existential distress Recurrent heart failure admission Increasingly frequent appropriate defibrillator shocks Possible defibrillator deactivation or non-replacement Before ventricular device implantation or transplant referral When initiating palliative inotropic therapy Declining functional status due to progressive heart failure or comorbidity Patients and/or informal carers/surrogates disagree on goals of care Request for assisted suicide
